# Editorial: Industrial Biotechnology Forum (http://ibf-conference.org)

**DOI:** 10.3389/fbioe.2019.00434

**Published:** 2019-12-20

**Authors:** Thomas B. Brück

**Affiliations:** Werner Siemens Chair of Synthetic Biotechnology, Department of Chemistry, Technical University of Munich, Garching, Germany

**Keywords:** industrial biotechnology, bioeconomy and circular economy, bioprocess engineering, metabolic engineering, bioseparation technology, synthetic biology, biocatalysis

The Industrial Biotechnology Forum (IBF) on the 13–14.3.2018 was a showcase of international academic and international research activities covering broad areas of expertise encompassing enzyme catalysis, metabolic- and bioprocess engineering as well as downstream bio-separation engineering, synthetic biology, and bio-informatics trends in biotechnology. Not surprisingly the manuscript contributions submitted to the Frontiers IBF special issue covered various current topics on metabolic engineering and biocatalysis, bio-separation technology, and bioprocess engineering.

In that context, the fermentative production of amino acids and their functionalized derivatives constitutes a major industrial application, as it delivers sustainable building blocks for chemical and pharmaceutical synthesis under economic production constraints. Conventionally, amino acids are either generated in classical, recombinant microbial hosts such as *Escherichia coli* or *Corynebacterium glutamicum* using established glucose based fermentation media. However, glucose based fermentations are rather expensive compared to equivalent applications with alternative carbon sources, such as glycerol, which is considered to be a waste stream of biodiesel production processes. In that context, the featured manuscript in this IBF special issue presented by Mindt et al., described the efficient conversion of the sustainable and cost efficient carbon source glycerol into Methylglutamate using a metabolically engineered *Pseudomonas putida*. This work is remarkable as it expands the microbial chassis that can be applied to directly generate functionalized amino acids. In essence, *P. putida* is genetically traceable and has the inherent metabolic capacity to convert glycerol in value added products. As such this recombinant production system synergistically expands the available microbial host platforms to enable production of renewable amino acid based commodity chemical intermediates. With a reported product yield of 17.9 g l^−1^ N-methylglutamate in fed-batch cultivation mode, the reported process has a high potential for consequent technical validation and commercialization. Much like amino acids, aromatic amines are highly sought chemical building blocks in the manufacture of dyes, pesticides, drugs, polymers among other industrial products. To that end, the featured article by Nargesi et al. reports the *de-novo* engineering of a para-amino-L-phenylalanine biosynthesis pathway utilizing genetic elements of the fungal Ehrlich pathway. The construction of this synthetic biosynthesis route allows recombinant production of the aromatic amines para-amino-phenylethanol (PAPE) and para-amino-phenylacetic acid (4-APA) from glucose in *E. coli*. Under controlled fed-batch conditions the best recombinant production strains reached yields of 2.5 g l^−1^ PAPE (11% C mol mol^−1^ glucose) and 3.4 g l^−1^ 4-APA (17% C mol mol^−1^ glucose) from glucose respectively. These production levels are the highest obtained for any aromatic amine thus far.

Driven by current climate change concerns, there is an increasing scientific activity in the design- and industrial deployment of microalgae based photoautotrophic bioprocesses, that allow rapid CO_2_ fixation and its subsequent biomass based conversion into value adding industrial products. The high CO_2_ fixation rate is inherently linked to the enhanced photosynthetic efficiency of microalgae, which is about twice as high as those of equivalent vascular, terrestrial plants. Moreover, microalgae can be cultivated on marginal lands using waste-, brackish-, or salt water. Therefore, algae cultivation does neither compete with agricultural activity nor rely on freshwater resources, that are essential to maintain terrestrial plant-, animal- and human populations. The excellent review by Molazadeh et al. links and reviews bioprocess for algae based CO_2_ fixation with the algae's ability to remove excess nutrients, such as nitrogen and phosphorous, from agricultural- and municipal wastewater streams. For each of the featured bioprocess options, the article covers details of energy efficiency and performance differences across microalgae species. In its conclusion the article suggests that linking algae biomass growth with wastewater treatment could be the key to resolve economic and ecological barriers that currently hamper industrial deployment of algae based biofuel solutions. While algae cultivation processes are reaching industrial maturity, harvesting and downstream processing of algae biomass from aqueous media remains a scientific and economic challenge. This holds particularly true for biomass separation from highly saline aqueous solutions due to the charge difference between algae biomass and the cultivation medium. In that context, the innovative article by Bracharz et al. describes a new, energy efficient flocculation method. Here a pH shift in combination with the first in class use of a tannin based flocculent, provides a mass efficient algae biomass dewatering process. Moreover, the utilized tannin based flocculent, is derived from a side stream of a tea leave extraction process and is therefore not only cost efficient but also completely biodegradable, which renders this new algae dewatering process highly economical and ecologically efficient.

The example of algae biomass harvesting demonstrates that efficient bio-separation technologies are at the heart of an economical bioprocess design. The topic of bio-separation is explicitly highlighted in the review of Schwaminger et al. This review focuses specifically on the rather new and highly specific technology for bio-product purification by the aid of magnetic beads from complex biological matrices. The review (Schwaminger et al.) provides an overview of the progress of capture and separation of biomolecules derived from biotechnology and food technology. While several technological challenges, such as development of flexible separation devices and more broadly applicable separation conditions have to be resolved, it is apparent that magnetic separation technologies will increasingly be applied in industrial bioprocesses.

In that respect, the IBF special issue highlights innovations in up- and downstream bioprocess development that will capacitate industry and society alike to establish a globally sustainable bioeconomy in order to reclaim, preserve and protect the natural resources of our planet.

## Author Contributions

TB has orgnized and edited the Frontiers special issue Industrial Biotechnology Forum 2018. Additionally, he wrote the editorial of this special issue.

### Conflict of Interest

The author declares that the research was conducted in the absence of any commercial or financial relationships that could be construed as a potential conflict of interest.

